# Return to work after sick leave due to mental illness - a qualitative study on the perspective of workplace integration managers

**DOI:** 10.1007/s00127-025-02906-3

**Published:** 2025-04-30

**Authors:** Anna Pelizäus, Martina Geipel, Johannes Hamann

**Affiliations:** 1 Department of Psychiatry and Psychotherapy, TUM School of Medicine, Technical University of Munich, Ismaninger Straße 22, 81675 Munich, München, Germany; 2 Department of Psychiatry, Psychotherapy and Psychosomatic Medicine, University of Augsburg, Bezirkskrankenhaus Augsburg, Geschwister-Schönert-Straße 1, 86156 Augsburg, Germany; 3 Bezirksklinikum Mainkofen, Mainkofen A 3, 94469 Deggendorf, Germany

**Keywords:** Qualitative research, Thematic analysis, Return to work, Mental disorders, Workplace, BEM manager

## Abstract

**Background and aim:**

Increasing numbers of people are unable to work due to mental illness and this is an increasing problem on both a personal and societal level. In Germany, a workplace integration management system (BEM) has been legally required since 2004 to support return to work (RTW). However, its uptake and success, especially regarding mental illnesses, is still unclear. This study was conducted to identify the current state of RTW after episodes of mental illnesses from the perspective of workplace integration managers and explore potential barriers and facilitators that influence the course and outcome of the BEM process.

**Methods:**

Semi-structured interviews with BEM managers (*N* = 14) from the greater Munich area were performed and analyzed using Thematic Analysis.

**Results:**

In their work, BEM managers tend to find themselves in a field of tension between the personal concerns of returning employees and the employer’s business interests. They experience mistrust and lack of openness on the part of the returnees, while employers show little willingness to fully invest in the process. Lack of or incorrect information about BEM and on mental illness seems to promote these disruptive factors, as well as others.

**Conclusion:**

Broad education on BEM appears to be a promising means to reduce fear among returnees and to better reach the processes potential. In addition, a more open approach to mental illness could simplify the process for all involved.

## Background

Absenteeism due to mental illness reached a new high in Germany in 2022 [1]. Mental illnesses were the third most common cause of incapacity to work and the duration of absences tended to be long [2]. However, more than temporary incapacity to work is at stake, as mental illness is the main reason for receiving a pension due to reduced earning capacity, as figures from the German pension insurance scheme show [3]. This leads to an immense financial burden for health insurance funds and companies [4].

The loss of the ability to work is also a problem at the individual level as the importance of work goes beyond its purely monetary aspect. Work can contribute to health and provide a social identity by giving people a sense of being needed and of actively contributing as a part of society [5]. Having a paid job can even lead to a decrease in symptoms and reduce the rate of rehospitalization [6]. In addition, having permanent employment in the primary labor market can also significantly improve quality of life [7]. A positive prognostic factor also seems to be an early start for work in the sense of supported employment [8]. A (temporary) loss of the ability to work is a negative prognostic factor.

Regarding RTW, it is worrying to observe that this ideal often fails. In 2020, almost 800,000 inpatient treatment cases were documented in psychiatric and psychosomatic clinics in Germany [9]. A survey conducted in the greater Munich area identified that only 20% of all inpatients had a current employment contract. Of these, only about two-thirds returned to work (at least temporarily) after treatment [10].

The causes of this imbalance can be assessed in many ways, but an exhaustive treatment is certainly not exhaustively possible in this context. Fears in relation to the workplace are mentioned, as well as preexisting conflicts with colleagues or managers [11]. Furthermore, from the patient’s point of view, there often seems to have been insufficient support from the employer when the old job is returned to [10].

The importance of RTW for the individual and society is just as obvious as the fact that RTW as a goal often seems to be inadequately achieved. Internationally this issue has been addressed by many researchers and there is a wide range of different interventions designed to support the RTW. Amongst others many of these interventions address the employers’ side. However since these interventions differ greatly in their implementation and objectives (because most of them heavily depend on the country specific background regarding legislation and health system, it is difficult to derive generally valid recommendations for action [12].

### Workplace integration management in Germany

To systematically support RTW, workplace integration management (BEM) was legally established in Germany in 2004. The goal of BEM is the fastest possible RTW for employees with all types of health problems, as well as the long-term securing of the ability to work despite restrictions. The employer is responsible to offer a BEM process to all employees who have been on sick leave for six weeks within a year. Therefore, a written invitation is being sent to the respective employee including information about the procedure and goals of the BEM process as well as about its voluntary nature and confidentiality. If the employee agrees to the process a plan of action is determined in subsequent discussions with different stakeholders. Apart from to the BEM manager, who carries out and coordinates the process, a member of the works council, the company doctor, or even a person whom the employee trusts, may be involved. The position of the BEM representative can be held by any member of the company or an external provider. Discussions can lead to various interventions, from workplace adjustments to a transfer of position. The measures agreed upon are to be implemented promptly, and their effectiveness should be reviewed repeatedly. Ideally, the BEM process should take the form of an open-ended search process, characterized by active cooperation of all the actors involved, from which both the employee and the employer ultimately benefit in the end [13].

The high number of those without any RTW after a longer absence shows that this ideal image of the BEM process is often not achieved. The aim of the present study was to identify the current state of RTW in Germany after episodes of mental illnesses from the perspective of BEM managers and explore potential barriers and facilitators that influence the course and outcome of the BEM process.

## Materials and methods

We chose a qualitative approach for our study, as the aim was to portray the experiences of BEM managers, and what they perceive as facilitators and barriers concerning the BEM process, in an interpretative way and not to examine existing hypotheses. Our data analysis was based on Braun and Clarke’s Thematic Analysis, a method that allows both inductive and deductive access to patterns and themes within a data set [14]. In this analysis, a more inductive approach was chosen, as we intended to derive hypotheses from our findings. At the same time, there was a deductive component, since we assumed that the participants would report on facilitators and barriers that they perceive.

To ensure the quality of all stages of our qualitative study, we used the Consolidated Criteria for Reporting Qualitative Research checklist [15].

### Recruitment and participants

Potential candidates were BEM managers identified within the contact network of the RETURN study [16] or among the first author’s acquaintances. In all cases, the first approach was made via email. The message included basic information on the study’s aims and the fact that it is being carried out as part of a doctoral thesis. The participation’s voluntary nature and its confidentiality were also communicated. The recruitment criteria in this purposive sample were the participants’ function as BEM managers and their experience with reintegrating (mentally) ill employees.

As we began the data analysis in parallel with participant recruitment, we ended the recruitment phase as soon as we considered the data to be saturated and no further topics could be identified.

### Interviews and data collection

For demonstration purposes, the first interview was conducted by J.H., a male professor of psychiatry and psychotherapy, accompanied by A.P., a female medical student who was then inexperienced in the field of qualitative research. The following 13 interviews were performed by A.P. herself.

Semi-structured interviews were conducted either at the BEM managers’ workplaces or via video or phone calls when required by the company’s COVID-19 restrictions. A recording device was used to capture the interviews for later transcription.

Each interview began with the interviewer asking the participant to relate their professional background and their individual experience with the BEM process. This included information on the processes monitored per year and on the number of employees who fall within the BEM manager’s remit. In the following, the participants were asked a set of open questions that were developed before the interviews: *(a) How is your experience with the reintegration of mentally ill employees? (b) How is the return-to-work process structured in your company? (c) Is the duration of the sick leave of importance? (d) How do the mentally ill returnees go into the BEM-talks? (e) How is mental illness viewed in your company? (f) In general*,* do you personally consider work to be good or damaging for mental health?* Over the course of the study, a new question was added to replace question (c), as it produced significantly less content than the other questions. For this purpose, a theme was drawn from an aspect mentioned repeatedly in earlier interviews: *Is there contact with psychiatric clinics or practitioners during the process and what does this look like?* Because each topic contained several subtopics, additional questions were made available for further exploration. The interview’s duration was determined by the participants’ motivation to talk about the subject and by their personal experience. As a result, the length of the interviews varied from 26 to 68 min. In no case were interviews repeated or supplemented afterwards.

### Data analysis

Each interview was transcribed verbatim. The cited passages were translated from German for this document. The analysis of the transcripts took place in several steps (familiarization with data, initial coding, finding themes, reviewing themes, defining themes, write-up) as conceptualised by Virginina Braun and Victoria Clarke in their publication on Thematic Analysis [14]. Thus, after A.P. had repeatedly read and familiarized herself with the raw data the software MaxQDA was used to assign codes (“initial coding”) to all content that appeared relevant in relation to research questions (= state of the BEM process from the perspective of BEM managers and barriers and facilitators). The lengths of codes varied from a few words to whole sections of text. In the next step (“finding themes”), mind maps and provisional visualizations were used to relate the initial codes to each other and develop higher-ranking codes and overall themes. For example, as expected, the hierarchisation and linking of the codes actually made it possible to create an overall theme with facilitators and barriers. Other themes, such as the knowledge theme (see result section), arose inductively when looking at the code systems. While looking for themes, overlaying codes were revised to ensure the quality of the analysis, whereby some overlaps can be useful, as “overlaps are partly how patterns are formed” [17]. The themes found were repeatedly compared with the corresponding text passages to ensure that each theme tells a coherent story about the underlying data (“reviewing themes”). Finally, for the categories that passed scrutiny, appropriate quotes reflecting the core content were selected to represent the research findings (“defining themes” and “write-up”). Furthermore, throughout the data analysis, codes and text passages were repeatedly discussed in group sessions with experienced (J.H.) and inexperienced (M.G., a medical student) qualitative researchers.

Due to the word count limitation, in the results section, only the most prominent themes (facilitators and barriers, knowledge of BEM and knowledge of mental illness) and codes, as well as the associated quotations, are presented.

## Results

The 14 participating BEM managers were 9 women and 5 men (aged between 30 and 60 years) working at companies in the Munich area. Within their area of responsibility, they had between 40 and 14,000 employees and dealt with an average of 95 cases per year. Because no inclusion criteria were set on the participants’ professional background, there was a large variety of previous experience. Some of them took on their role while employed at the HR department, while others held the position of BEM manager parallel to their regular function in another field of work. One of them carried out BEM as her main activity, as part of her work in occupational health care. These differences led BEM managers to differ in the extent to which BEM activities were part of their daily working lives.

The interview guide’s questions focused on the experiences of the BEM managers with mentally ill returnees during the BEM process. The answers to questions (a) and (d) took up most of the conversation and brought up the most content. During the analysis, we were able to identify the overarching themes of facilitators and barriers, which differ for the three actors described and each consist of a pattern of codes or superordinate codes that can be interpreted as attitudes, behaviors or circumstances that influence the process and the outcome either in a positive or negative way (Table 1). The frequency of the corresponding text passages are given in brackets. The most frequent factors are presented below in the text.

**Table 1:** Facilitators and barriers in the BEM process

Mentally Ill Returnees	Employer	BEM Manager
Facilitators ● Openness about one’s own mental illness **(25)** ● Cooperation and personal responsibility **(13)** ● Humility and gratitude **(10)** ● Self-acceptance **(5)** ● Self-awareness in terms of one’s own limitations **(2)** ● Reasonable expectations **(2)**	Facilitators ● Willingness to fully engage in the process **(42)** ● Flexibility and willingness to compromise **(19)** ● Trusting atmosphere **(19)** ● Awareness of mental illness **(17)** ● Supportive attitude **(13)**	Facilitators ● Practical support **(21)** ● Mediating **(19)** ● Emotional support **(15)** ● Providing information **(13)** ● Prior knowledge of mental illness **(13)**
Barriers ● Distrust **(39)** ● Lack of openness **(24)** ● Unreasonable expectations **(24)** ● Lack of self-acceptance in terms of one’s own limitations **(15)** ● Lack of personal responsibility **(14)** ● Fear of former trigger factors **(9)** ● Fear of stigma **(9)** ● Self-stigmatization **(4)** ● Feelings of guilt **(4)**	Barriers ● Blockage of a smooth process **(21)** ● Fears of contacts **(18)** ● Lack of acceptance in the team **(18)** ● Business focus **(16)** ● Preexisting conflicts with the manager **(6)**	Barriers ● Distinction from a therapist’s role **(16)** ● Fears of contacts **(9)** ● Emotional involvement **(5)** ● Desire for more knowledge about mental illness **(5)**

### Mentally ill returnees in the BEM process

BEM managers remarkably often reported an attitude characterized by **distrust**. A considerable proportion of mentally ill returnees seemed to assume that the process was directed against them and reacted accordingly with uncertainty and negative behavior.

“Nothing happens because they are simply of the opinion: ‘No, I feel bullied. I feel like I’m being bullied, I don’t know what, and you just want me out of here anyway’ and stories like that come up. So it’s not always that easy.” (Participant 10)

“They don’t see it as integration, but more as exclusion.” (Participant 14)

This finding goes hand in hand with a **lack of openness**. Yet **openness about one’s own mental illness** (and the associated limitations) is an important factor for a successful BEM process, as was repeatedly emphasized by the BEM managers. It makes it easier to find measures and leads to better acceptance among managers and colleagues during RTW.

“And when someone comes in with his illness, it would be important to me that he simply says what his limitations still are, so that we can really find the appropriate measure with which the BEM then tries to make it easier for him to return to work. (…) It doesn’t matter to me which diagnosis is behind it, what he actually had, whether it’s panic attacks or just- The medical stuff doesn’t interest me. I’m really interested in people being open about ‘what can I do again?’” (Participant 9)

It should be briefly noted that many factors were identified during the analysis to condition the **openness** of returnees. The most important are trusting atmosphere of conversation and a trusting relationship at the workplace.

### The role of the employer as responsible “pacemaker”

BEM managers often mentioned how important it was that they were **willing to engage in BEM as a process**. This included not only the early initiation of the process in view of its relevance, but above all the willingness to proceed patiently and thus give time and space for finding results and development.

“A great wish, of course, would be that the acceptance of the healthy, especially of the decision-makers, would be so great that they would say ‘Ok, uh, I have an employee here who is now suffering from an illness. The healing process takes longer than six weeks, but I am ready to reintegrate him completely’.’” (Participant 5)

A similarly strong impact, albeit in a negative sense, appears to occur if the employer **blocks the smooth running of the BEM process**, for example by appointing an inexperienced BEM representative. As the law does not clearly define who is to lead the BEM, this task may be assigned to a person who is somewhat or completely unfamiliar with the procedure.

“Because the BEM is carried out by our managers with the particular employee, we simply received several reports from various sides that the BEM is often not of the required quality. Yes, of course, the managers are not trained. It doesn’t have good quality, it is too often terminated early,it doesn’t take place at all. So, it depends a lot on the manager.’” (Participant 9)

### How BEM-managers perceive their position

The BEM managers play a special role in our analysis, as they themselves are active in the BEM process and in fact evaluated their own work themselves during interviews. Instead of speaking about supporting attitudes and behaviors, as was done when we gave an account of employer and employee sides, the BEM managers mainly described their actual work, which in part also reflects the understanding they themselves have of their role. For example, they see it as their duty to offer **practical support** on work-related issues and to **provide information**. Although **mediation** and **emotional support** also play a major role as tasks, there was a clear **distinction in terms of the perceived therapist role**. This included, on the one hand, the feeling of being wrongly pushed into this role by the returnees and, on the other hand, self-reported helplessness regarding helping employees with mental illness.

“I’m not a psychologist and I can’t do anything about his illness and I can’t help him. I offer support at the work level so that he can plan his return and feel supported, and everything else must be done by his therapists. So it is also important that this is clear from the beginning.” (Participant 3)

The BEM managers find themselves in an area of tension: in the conversation opposite them is a person whose problems need to be addressed, while at the same time, the BEM serves to find solutions that are expected by the employer.

“So you have to be careful not to cross this line, because when employees say, ‘I find the talks with you so good and it does me so much good,’ then I also say, ‘Doing good is one thing, but it also has to lead to a goal. How can the goal be if you only come to me? We must have some kind of solution then.’” (Participant 14).

### Knowledge gaps in the context of the BEM process

**Fig. 1:**
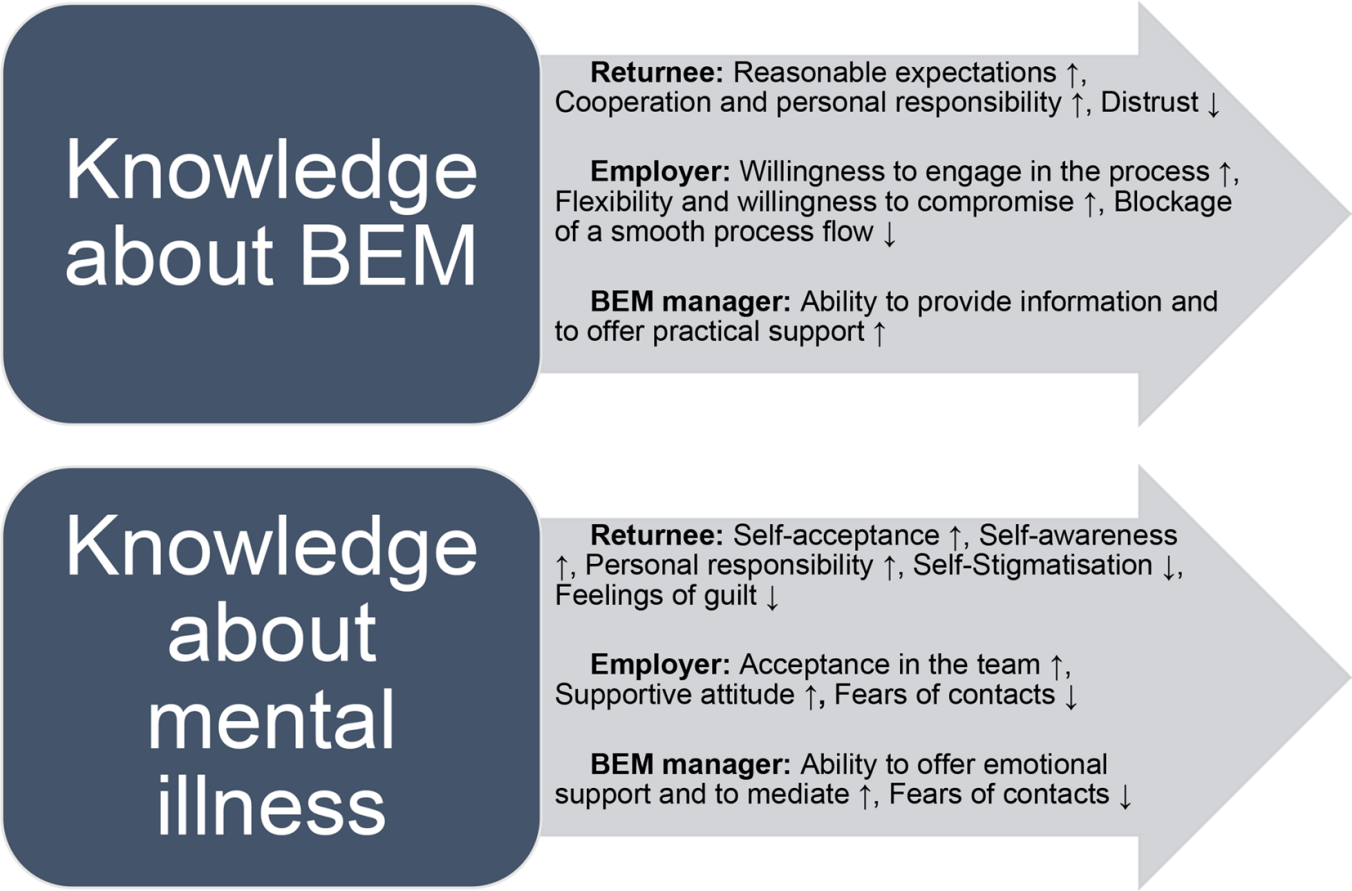
How knowledge strengthens facilitators (↑) and reduces barriers (↓) of the BEM process

It became clear that knowledge about BEM and about mental illness function as overarching concepts, as many of the facilitators and barriers found can be subsumed under them. Knowledge seems therefore to be a decisive factor in successful BEM processes. This observation can be made in relation to all three main actors as shown in Fig. 1, in an illustration that is intended to show how knowledge of the objectives and procedures of BEM (in short: “knowledge about BEM”), as well as knowledge about and understanding of (one’s own) mental illness (in short: “knowledge about mental illness”) are linked to the categories given in Table 1. Several facilitators were reported to be reinforced (↑) by knowledge, whereas barriers were reduced (↓). The following describes how selected facilitators and barriers fit into the concept of knowledge or non-knowledge. For reasons of space, not every point can be supported by a quotation.

Mentally ill returnees who do not know what the BEM process is about could have an **unrealistic attitude of expectation** and show a **lack of responsibility**.

“There are also employees who really have this mentality that you have to ask them to everything and they don’t have to move in their behavior. Most of the time it is like this that both sides, the company and the employee, are required to move somewhere toward a common point and sometimes it is also the case that the employee says ‘I am sick, I can’t help it. Now please solve my problem’ and then it falters.” (Participant 11).

Such employees might also show **mistrust** because they likely do not know what to expect.

On the employer side, the level of understanding of the BEM process is also reflected: those who know enough about BEM and what it should achieve are **willing to invest in the process and its measures**, while in the opposite case, **the process tends to be blocked** because it seems superfluous.

Third, a BEM manager that is sufficiently familiar with the process can provide more targeted **support and information**.

However, knowledge and ignorance play a role in relation to BEM per se as well as in relation to mental illness. If returning employees understand their mental illness and its impact on their ability to work, this increases, for example, **self-acceptance** and the **ability to communicate the resulting limitations**.

“Sometimes it is a process over the period of time that you have to reassess your self-image, the image of others, what, no, is at all possible and what you would like to be able to achieve, but which is perhaps no longer possible due to the limitations. This is often very noticeable in the psychological area, yes, that of course the question is ‘Does the person see his or her resilience realistically?’” (Participant 3)

A lack of understanding can lead to anxiety and **self-stigmatization** and a **lack of personal responsibility**.

On the part of the employer and the BEM manager, the shortage of knowledge about mental illness manifests itself primarily in the **fear of contact**. Knowledge of mental illness, on the other hand, was described as positive. Not only in terms of a **supportive attitude** toward the employee but also regarding greater **acceptance in the team**.

“I think the most important factors are actually to sensitize the employees and the managers to this and to get an understanding of ‘What’s the problem? What does such an illness do to the employee? And how can we support them to prevent this?’ In other words, to prevent these trigger points.” (Participant 11)

Finally, the BEM manager himself or herself benefits from it: whoever is more familiar with what mental illness means can **mediate** better between the parties involved and provide **emotional support**.

### From inpatient treatment to the BEM process: a rather unexploited opportunity

The theme of knowledge and non-knowledge also influences the BEM process beyond the main stakeholders. The BEM managers reported that they have only a few contacts with psychiatric clinics and treatment providers, and the clinicians there often do not seem to want to be contacted. This is not the case for the BEM managers, who explicitly called for more exchange of information.

“Um, yes, I would find it useful to have such a better connection, so if it is simply the normal doing, um, um, therapist and company connect in this process, then I would consider that very, very useful, but I rather have the feeling that it comes from the other side, um, that it is not so desirable.” (Participant 11)

The participants also expressed the wish for the psychiatric patients should be informed of the background of BEM before they interact with the process for the first time. Some consideration of what the returnee can currently achieve should have also been provided during treatment.

## Discussion

This study explored experiences of BEM managers with the RTW process of mentally ill employees and identified facilitators of and barriers to successful RTW processes (Table 1) that are often difficult to reconcile.

In the following section, we focus on our key findings and discuss them in relation to their potential relevance to practice and in the context of previous research.

Openness on the part of mentally ill returnees appears as much of a central facilitator for the BEM process as lack of openness is of a barrier. The BEM managers in our study see a longer-term advantage to disclosure in the workplace and during the BEM process, which is why they would explicitly want the returnees to disclose to be able to perform the BEM processes successfully. Disclosure facilitates the mutual identification of measures and the RTW of the mentally ill employee in general, as openness can generate understanding among colleagues and managers. In other words, the openness that is desired does not concern naming the diagnosis per se but rather making the limitations caused by the mental illness obvious, so that all participating actors can deal with them. However, such openness is of course also associated with the fear and risk of stigmatization or disadvantage [18–19], which makes the frequent lack of openness on the part of the returnees logical. Openness in the sense of disclosure management seemed to be a very complex topic in the context of this study, which is in line with the results of earlier publications [20]. Due to the frequent thematic focus on openness in our study, we believe that it would be useful to explore the extent to which disclosure can be promoted in more depth to exploit its benefits for a successful BEM process.

Particularly striking was how far lack of knowledge, both about BEM itself and about mental illness, influences the BEM process. Insufficient information has been reported as linked to various barriers (e.g., mistrust, lack of openness, or fear of touch). This suggests that an increased awareness of BEM and an open approach to mental illness could be decisive factors in improving the process. In addition, it might lead to a more consistent implementation in the companies and a higher acceptance rate among employees. The adoption rate was not recorded in our study, but it has been shown that BEM is generally not always accepted [21].

Another key finding of our study relates to the knowledge transfer in the psychiatric treatment setting. The importance of knowledge about BEM and mental illness makes clear the practical relevance of this finding. However, the conditions for knowledge transfer during inpatient treatment are created, as the importance of RTW has found its way into German treatment guidelines [22]. Therefore, there should be more of a focus on actual testing and implementation of appropriate measures. It has already been shown that a more targeted preparation for RTW could promise superior outcomes for certain patient groups [23]. Likewise, in Sweden, the use of digital RTW tools has shown promise [24] and could provide an alternative means of education. Another means of facilitating communication and close knowledge gaps in both areas (BEM and mental illness) could be found in the increased involvement of occupational physicians, who can play a mediating role due to their unique expertise in medicine and its interface with the world of work [25]. The extent to which knowledge transfer could effectively lead to a higher return rate must be further investigated, although contradictory results have already been published. A German intervention study that investigated a psychoeducation model education, including information about the BEM process (along with group exercises on how to deal with colleagues during RTW, etc.), showed an improvement in perceived competence but not in terms of the actual return rate [26].

### Limitations of the study

The diversity and complexity of the codes found limited the range of discussion in this study. Because the study aim was to identify areas for improvement, the focus was on aspects that were not functioning well. Thus, the description of the participating actors does not provide a complete representation of reality. As participation in the study requires a certain commitment, the question whether there was selection bias among the participants should not be neglected. In fact, a certain bias can be assumed, as BEM managers are located organizationally on the employer’s side. In addition, the extent to which the interview situation (over telephone or Zoom call) may have distorted the interview content is unclear. We have used the Consolidated Criteria for Reporting Qualitative Research checklist [15] in the planning and execution of the study in order to maximise trustworthiness and credibility from the initial phase. However, the controversially discussed concept of intercoder reliability [27] was not applied because the data set was mainly coded by A.P. Furthermore, the transferability may be considered restricted as the BEM system is exclusively implemented in Germany.

## Conclusion

The BEM process, which usually occurs at the interface between inpatient stay and RTW, proved to be a promising but not yet optimized way of supporting employees following mental health-associated sick leave. Despite its limitations, our study was able to identify a variety of starting points (e.g., promoting disclosure or knowledge transfer) where adjustments could be made and where further in-depth research could begin.

## Data Availability

The dataset is not publicly available due to its confidential nature.
